# The Loss of Polysialic Acid Impairs the Contractile Phenotype of Peritubular Smooth Muscle Cells in the Postnatal Testis

**DOI:** 10.3390/cells10061347

**Published:** 2021-05-29

**Authors:** Nadim E. Hachem, Luisa Humpfle, Peter Simon, Miriam Kaese, Birgit Weinhold, Juliane Günther, Sebastian P. Galuska, Ralf Middendorff

**Affiliations:** 1 Department of Anatomy and Cell Biology, Medical Faculty, Justus-Liebig-University, Aulweg 123, 35385 Giessen, Germany; nadim_hachem@hotmail.de (N.E.H.); luisa.humpfle@med.uni-giessen.de (L.H.); 2 Institute of Biochemistry, Medical Faculty, Justus-Liebig-University, Friedrichstr. 24, 35392 Giessen, Germany; peter-simon@live.de (P.S.); miriam.kaese@gmx.de (M.K.); 3 Institute of Clinical Biochemistry, OE 4340, Hannover Medical School, Carl-Neuberg-Str. 1, D-30625 Hannover, Germany; Weinhold.Birgit@mh-hannover.de; 4 Institute of Reproductive Biology, Leibniz Institute for Farm Animal Biology (FBN), Wilhelm-Stahl-Allee 2, 18196 Dummerstorf, Germany; guenther.juliane@fbn-dummerstorf.de

**Keywords:** polysialic acid, testis, smooth muscle cells, postnatal development

## Abstract

In the testis, the germinal epithelium of seminiferous tubules is surrounded by contractile peritubular cells, which are involved in sperm transport. Interestingly, in postnatal testis, polysialic acid (polySia), which is also an essential player for the development of the brain, was observed around the tubules. Western blotting revealed a massive decrease of polySia from postnatal day 1 towards puberty, together with a fundamental reduction of the net-like intertubular polySia. Using polysialyltransferase knockout mice, we investigated the consequences of the loss of polySia in the postnatal testis. Compared to postnatal wild-type animals, polySia knockouts showed slightly reduced smooth muscle actin (SMA) immunostaining of peritubular smooth muscle cells (SMCs), while calponin, marking more differentiated SMCs, dramatically decreased. In contrast, testicular SMA and calponin immunostaining remained unchanged in vascular SMCs in all genotypes. In addition, the cGMP-dependent protein kinase PKG I, a key enzyme of SMC relaxation, was nearly undetectable in the peritubular SMCs. Cell proliferation in the peritubular layer increased significantly in the knockouts, as shown by proliferating cell nuclear anti (PCNA) staining. Taken together, in postnatal testis, the absence of polySia resulted in an impaired differentiation of peritubular, but not vascular, SMCs to a more synthetic phenotype. Thus, polySia might influence the maintenance of a differentiated phenotype of non-vascular SMCs.

## 1. Introduction

The testis is responsible for the production of male germ cells and testosterone [1]. The major components of the testis are the seminiferous tubules, with their germinal epithelium [1] and the surrounding peritubular lamina propria [2,3,4]. In rodents, this structure consists only of a single layer of contractile (myoid) cells. These cells are suggested to affect both transport of spermatozoa and germ cell maturation, in addition to further paracrine and immunological functions [3,4]. Among seminiferous tubules, the so-called interstitial tissue is visible, which includes the testosterone-producing Leydig cells, blood vessels, immune cell populations and connective tissue components [5]. The entirety of the tubules is surrounded by the capsular tunica albuginea, which also comprises contractile cells [6,7].

Interestingly, during postnatal development, the contractile areas of the testis exhibit high amounts of polysialic acid (polySia) [8], which is an essential biomolecule for the development of the brain [9,10,11,12]. This linear polymer consists of sialic acid residues, and the degree of polymerization (DP) can reach more than 100 [13,14,15]. The polySia chains are highly negatively charged, since sialic acids contain a carboxyl group at C1 [16,17]. In mammals, polySia chains can be synthesized by two polysialyltransferases, ST8SiaII and ST8SiaIV [8,18,19]. During the postnatal development of the male reproductive tract, ST8SiaII and ST8SiaIV are expressed [8].

In addition to the reproductive tract and the brain, polySia also appears during the development of other organs, such as the heart, lung, kidney, and liver [20,21,22,23,24]. It seems to be that the loss of one of the polysialyltransferases can be partly compensated by the other one, since knockout mice without ST8SiaII or ST8SiaIV show only a relatively mild phenotype [25,26]. Additionally, in fish, a loss of one polysialyltransferase seems to be compensated, since in the course of evolution, several teleost lineages lost one of the polysialyltransferases [27,28]. In contrast, the simultaneous loss of both polysialyltransferases leads to remarkable neurodevelopmental alterations and higher postnatal mortality rates in double knockout mice [9,10,11,12]. In addition, polySia negative mice showed a reduction of fatty tissue and limb muscles, which comes along with growth retardation [9]. However, in other organs, such as heart, lung, stomach, pancreas, gut, spleen and kidney, no significant pathological changes were observed [9]. Regarding the male reproductive tract, the biological role and impact of polySia on the formation of the contractile areas are unknown so far.

The best-known cellular functions of polySia are mainly related to the modulation of cell–cell interactions and migration mechanisms [12,18]. Due to its negative charge, the interaction between adhesion molecules can be inhibited, resulting, e.g., in an increased migration capacity of polysialylated cells [29,30]. In addition to its impact on cellular interaction, polySia can bind several growth factors, such as brain-derived neurotrophic factor (BDNF), nerve growth factor, neurotrophin-3, neurotrophin-4, vascular endothelial growth factor, and basic fibroblast growth factor 2 (bFGF) [31,32,33]. The interaction can modulate the activity of such growth factors by altered sensitivity to proteases and/or binding efficiency with their respective receptors [31,32,34]. Thus, several cellular mechanisms can be modulated by polySia, which might also take place in polySia-positive areas of the testis during postnatal development.

To examine the impact of polySia on the contractile elements of the testis, we investigated the phenotype of smooth muscle cells (SMCs) in polySia knockout mice during postnatal development, demonstrating that the phenotype of peritubular SMCs, different to vascular SMCs, changed in the absence of polySia.

## 2. Materials and Methods

### 2.1. Animals

For developmental studies, testicular tissues were obtained from mice (C57Bl/6J) of different ages (postnatal day 1,4,7,10,15,20 and 25) housed in the animal facility of Justus-Liebig-University Giessen, Germany (8). Housing, animal care, and all procedures were conducted according to the guidelines for animal care and approved by the committee for laboratory animals of Justus-Liebig-University Giessen (case numbers A38/2011_V54-19c2015(1)GI20/23 and A29/2009_V54-19c20/15cGI20/23).

For the knockout studies, testicular tissues from 5 wild-type (*st8sia2^+/+^; st8sia4^+/+^*), 5 polysialyltransferase-deficient (*st8sia^−/−^; st8sia4^−/−^*) and 3 *st8sia2^+/−^; st8sia4^−/−^* mice were investigated (age 9.5 days). Mice were backcrossed to the C57BL/6J genetic background. These animals were obtained from and hosted in the animal facility of the MHH (Hannover Medical School Hannover) under specific pathogen-free conditions. All protocols for animal use were in compliance with the German law for protection of animals and approved by the local authorities (33.12-42502-04-18/2932).

### 2.2. Immunohistochemistry

The testes were fixed in standard Bouin solution and embedded in paraffin (8). For immunohistochemistry, 5 µm sections of paraffin-embedded testis were used. The slides were dewaxed in xylene and rehydrated in a descending ethanol series. Resulting rehydrated tissue sections were incubated with the following primary antibodies: mouse anti-α-smooth muscle actin (SMA) monoclonal antibody (mAb) (Sigma-Aldrich, St. Louis, MO, USA; 2 µg/mL), rat anti-CD31 mAb (Dianova, Hamburg, Germany; 0.5 µg/mL), rabbit anti-calponin mAb (Abcam, Cambridge, UK; 0.134 µg/mL), rabbit anti-proliferating cell nuclear antigen (PCNA) mAb (Abcam, Cambridge, UK; 0.378 µg/mL), rabbit anti-PKG polyclonal antibody (pAb) (Enzo, Lausen, Switzerland; 2 µg/mL), and anti-polySia mAb 735 (10 µg/mL). Each staining (with the exception of CD31) was performed in a comparable way. The primary antibodies were always incubated overnight at 4 °C. Thereafter, sections were washed with PBS, and finally, a peroxidase-marked polymer kit (DAKO, Hamburg, Germany), together with DAB (3,3′-Diaminobenzidin) reagent, were used to visualize the selected antigens. For CD31 staining, goat anti-rat-IgG-biotin (Amersham, Little Chalfont, UK) and streptavidin peroxidase (Dianova) were applied, but not the polymer kit.

In the case of mAb 735, in parallel, sections were treated with endoneuraminidase (endoN) (10 µg/mL) overnight at 37 °C. EndoN cleavages polySia chains into oligomers consisting of up to 7 sialic acid residues [35,36,37]. However, the mAb 735 needs a minimum chain length of 8 for binding [38]. Thus, the combination of endoN and the mAb 735 is a powerful combination to control the immunostaining against polySia. The mAb 735 and endoN were provided by Martina Mühlenhoff (MHH, Hannover, Germany) [35,39].

All polySia slides were additionally counterstained with hematoxylin. Furthermore, slides were processed without a primary antibody. These slides were only treated with PBS/0.2% bovine serum albumin. Some of the polySia-stained tissues had already been used for the analysis of the polysialylation status in the epididymis and tunica albuginea testis [8]. All pictures were taken with a Zeiss Axioskop 2 plus (Carl Zeiss Vision, Munich, Germany) and processed with Axio Vison Software (Carl Zeiss Vision).

### 2.3. Western Blotting

For Western blotting, testis samples were homogenized in a lysis buffer, as described earlier [8] PolySia was visualized with the mAb 735 (1 µg/mL) [33,40,41]. An aliquot of each sample was treated with endoN (1 μg/mL, 4 h at 37 °C). The binding of mAb 735 was detected with HRP-conjugated antibodies (Dako, Hamburg, Germany) and a chemiluminescence Super Signal kit (Thermo Fisher, Kehl, Germany).

### 2.4. Measurement and Counting of the Proliferating Cells

Three different sections of each animal were immunostained with antibodies against PCNA, as described above. Using a measuring tool from AxioVision Software, the total area of the testis was determined in every section. To obtain the area of the seminiferous tubules, we subtracted the interstitial tissue from the total testis area. Using a corresponding tool from Image J 1.50e (public domain software, NIH, Bethesda, MD, USA), we counted the proliferating peritubular myoid cells. Thereafter, we calculated the ratio of counted peritubular cells per area of seminiferous tubules in each section.

### 2.5. Statistical Analysis

Data were analyzed with Graph Pad Prism 8.0 software using ANOVA and a multiple-comparison Tukey test. The values passed the Shapiro–Wilk normality test. Statistically significant differences are given the labels: *p* < 0.05 (*), *p* < 0.01 (**), *p* < 0.001 (***), and n.s. for not significant (*p* ≥ 0.05).

## 3. Results and Discussion

### 3.1. Loss of Polysia Leads to Altered Expression Patterns of Distinct Protein Markers of Peritubular SMCs in Postnatal Testis

**Figure 1:**
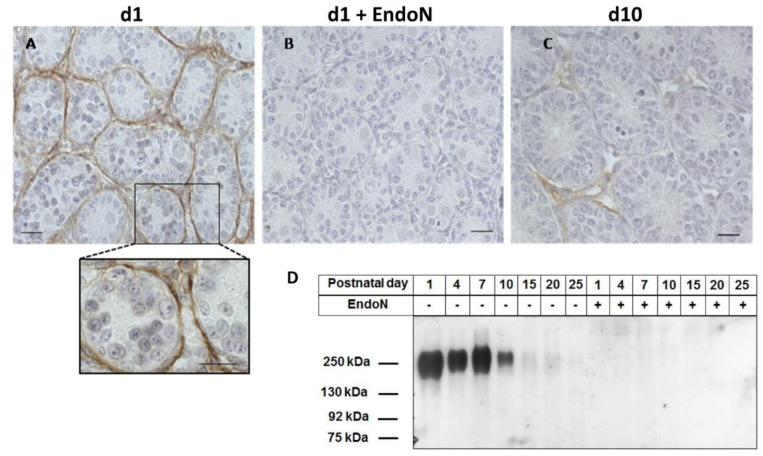
Visualization of polySia in postnatal testis of wild-type mice. (**A**–**C**) For the immunohistochemical localization of polySia in postnatal testis of 1- and 10-day-old mice, tissue sections were treated with a mAb against polySia. All scale bars: 20 μm. (**B**) For negative control, the tissue samples were pretreated with endoN to degrade polySia. The sections were counterstained with hematoxylin. (**D**) In addition, protein lysates were separated by SDS-PAGE (0.3 μg protein/lane) for Western blotting. The immunostaining was performed with an anti-polySia mAb. Samples treated with endoN were used as negative controls.

As previously described, in the postnatal murine testis, polySia-positive areas were found in the tunica albuginea [8]. In addition, strong peritubular staining (Figure 1A) occurred around almost all tubules at postnatal day 1. The degradation of polySia by endoN abolished the staining, verifying the specificity of the polySia visualization (Figure 1B). It is known that polysialylation decreases with increasing concentrations of collagen in contractile areas of the male reproductive tract and that polySia-positive areas are colocalized with SMA staining [8]. At postnatal day 10, only minor amounts of polySia were detectable (Figure 1C). In line with the immunohistochemical visualization of polySia, Western blot analyses confirmed a decrease of polySia during postnatal development (Figure 1D). The results suggest that the main postnatal polySia-dependent processes occur during the first week after birth.

Therefore, it was of interest to know the extent of testicular changes in mice lacking both polysialyltransferases (*st8sia2^−/−^*; *st8sia4^−/−^*) at postnatal day 9.5, i.e., directly after the first important week. For this, testis samples of double knockout mice were compared to the corresponding *st8sia2^+/−^*; *st8sia4^−/−^* and wild-type mice.

**Figure 2:**
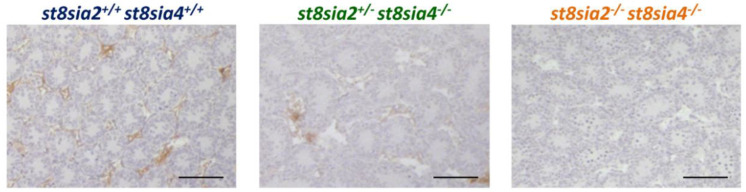
Visualization of polySia in postnatal day 9.5 testis of knockout and wild-type mice. For the immunohistochemical localization of polySia tissue sections were treated with a mAb against polySia. All scale bars: 100 μm.

In the first set of experiments, polySia was visualized demonstrating that the deletion of polysialyltransferases leads to reduced polySia levels in *st8sia2^+/−^; st8sia4^−/−^* mice and a complete loss of polySia in double knockout mice (Figure 2).

**Figure 3:**
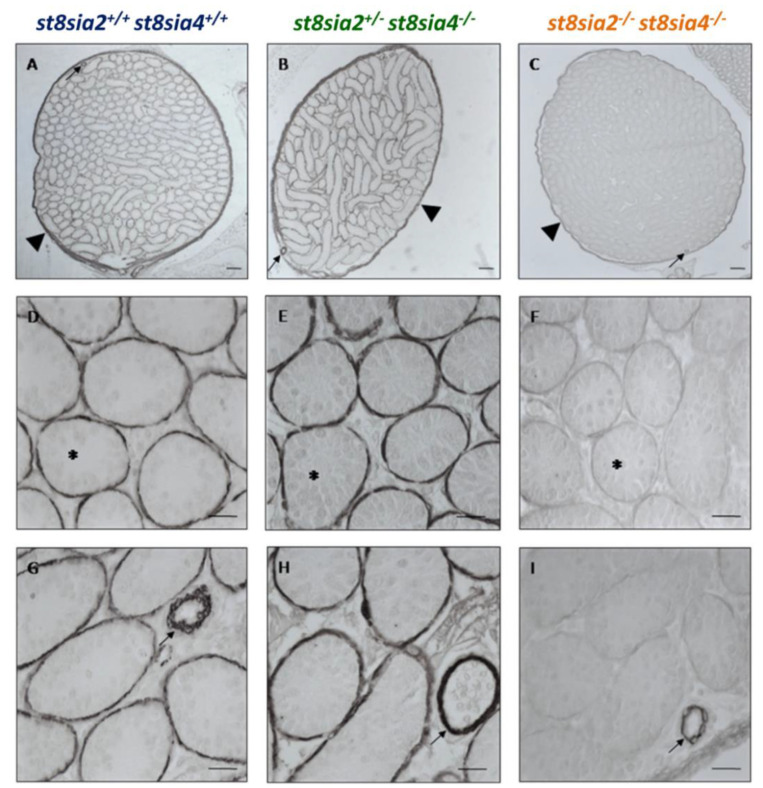
Immunohistochemical localization of SMA in testicular tissue of postnatal mice. The testicular tissue sections of postnatal wild-type (*st8sia2^+/+^*; *st8sia4^+/+^*), *st8sia2^+/−^*; *st8sia4^−/−^*, and polysialyltransferase-deficient (*st8sia2^−/−^*; *st8sia4^−/−^*) mice were stained with a mAb against SMA. (**A**–**C**) The tunica albuginea is labeled with a triangle, and selected tubules are marked with asterisks. Arrows indicate blood vessels. Scale bars: (**A**–**C**) 100 µm; (**D**–**I**) 20μm. Negative controls are provided in Supplemental Figure S1.

To analyze, if this loss of polySia affects peritubular SMCs, SMA was visualized by immunohistochemistry. In all genotypes, an unambiguous signal was located at the tunica albuginea (Figure 3A–C), as well as in blood vessels (Figure 3G–I). However, the SMA staining in peritubular SMCs seemed to be slightly stronger in wild-type and *st8sia2^+/−^; st8sia4^−/−^* testes in comparison to double knockout mice. The identification of blood vessels was confirmed by the endothelial cell marker CD31 (Supplementary Figure S2).

**Figure 4:**
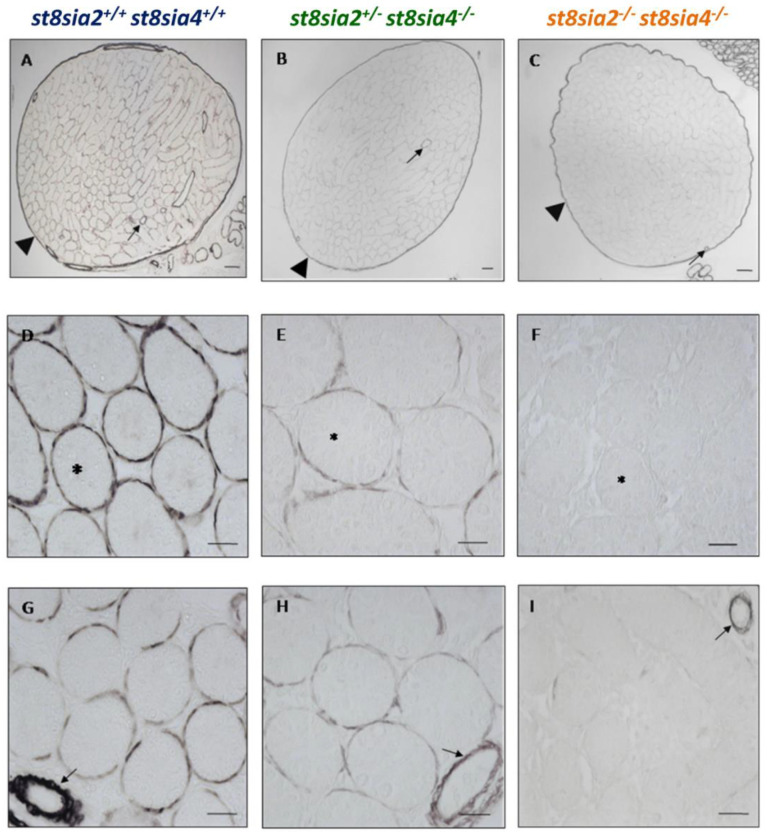
Immunohistochemical localization of calponin testicular tissue of postnatal mice. Using testis sections, calponin was visualized in postnatal wild-type (*st8sia2^+/+^*; *st8sia4^+/+^*), *st8sia2^+/−^*; *st8sia4^−/−^*, and polySia negative (*st8sia2^−/−^*; *st8sia4^−/−^*) mice. (**A**–**C**) The tunica albuginea is labeled with a triangle, and selected tubules are marked with asterisks. Arrows indicate blood vessels. Scale bars: (**A**–**C**) 100 µm; (**D**–**I**) 20 μm. Negative controls are provided in Supplemental Figure S1.

SMCs can be roughly classified in a synthetic and a contractile phenotype. With the development of the contractile machinery in SMCs, increasing numbers of contraction-specific proteins are expressed. Remarkably, SMA is expressed in all SMCs independently of the functional phenotype and is therefore used as a general marker of these cells. Thus, for a more detailed classification, more marker proteins have to be investigated. For this reason, calponin was used as further marker for muscle cells. In contrast to SMA, calponin is only expressed in more differentiated muscle cells [42,43]. As shown in Figure 4, the double knockout mice showed a dramatic decrease of the calponin expression in the peritubular muscle cells in comparison to the other two genotypes. However, calponin staining was unchanged in SMCs of the tunica albuginea (Figure 4A–C) and blood vessels (Figure 4G–I). Consequently, deletion of the polysialyltransferases comes along with a selective loss of calponin in the peritubular muscle cells. The results suggest that the dedifferentiation of SMCs is restricted to seminiferous tubules.

**Figure 5:**
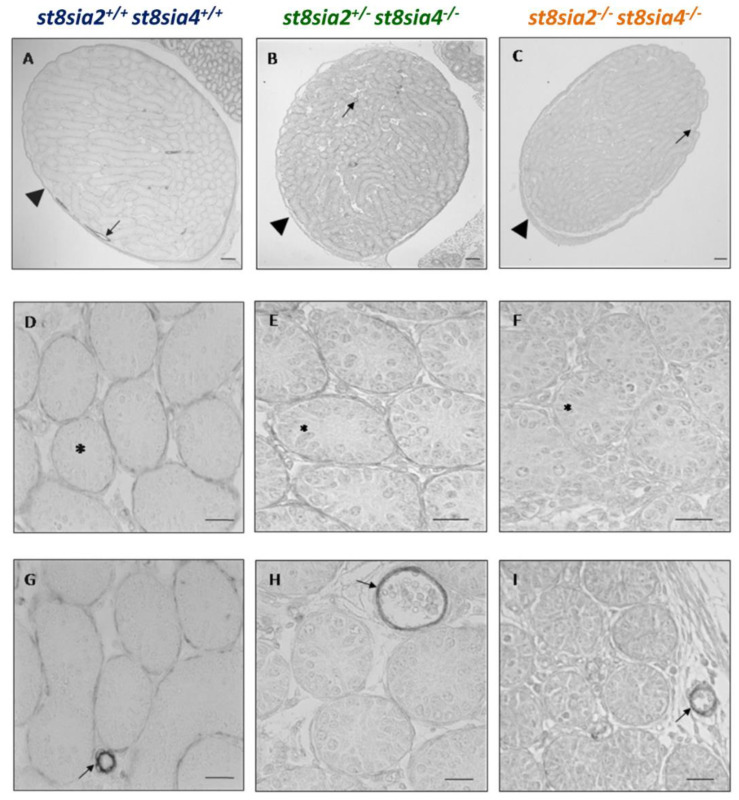
Distribution of PKG1 in testicular tissue of postnatal mice. For the immunohistochemical visualization of PKG1, testicular sections of postnatal wild-type (*st8sia2^+/+^*; *st8sia4^+/+^*), *st8sia2^+/−^*; *st8sia4^−/−^*, and polysialyltransferase-deficient (*st8sia2^−/−^*; *st8sia4^−/−^*) mice were treated with an anti-PKG1 pAb. (**A**–**C**) The tunica albuginea is labeled with a triangle, and selected tubules are marked with asterisks. Arrows indicate blood vessels. Scale bars: (**A**–**C**) 100 µm; (**D**–**I**) 20 μm. Negative controls are provided in Supplemental Figure S1.

In addition to the classical differentiation markers of SMCs, which predominantly belong to the group of structural proteins, we also tested PKG1 as a signaling molecule (Figure 5). PKG1 is an important regulator of smooth muscle cell relaxation [42,43]. Whereas PKG1 staining only showed minor alterations among the groups in vascular SMCs (Figure 5G–I), a dramatic loss of PKG1 in peritubular muscle cells was already detectable in *st8sia2^+/−^; st8sia4^−/−^* mice, and was completely absent in these cells in double knockout mice (Figure 5F,I).

Thus, loss of polySia coincides with decreased expression levels of proteins, which are involved in contraction and relaxation. Interestingly, the polySia binding partner bFGF represents a growth factor, which drives the differentiation of SMCs to a synthetic phenotype [43]. When bFGF is complexed by polySia, the direct binding of bFGF to its receptor seems to be inhibited resulting, for instance, in reduced cell growth [31]. Moreover, a loss of polySia may also alter the dynamic system of further growth factors in the postnatal testis, leading to the observed switch of SMC phenotype.

In light of the reduced numbers of SMCs with a contractile phenotype in polySia knockout mice, it is interesting to note that changes in the human peritubular lamina propria come along with disturbed spermatogenesis. In these patients, the peritubular areas become fibrotic, showing an accumulation of collagen in addition to decreased SMCs [2,4,44,45].

### 3.2. Peritubular SMC Proliferation Is Affected in polySia-Negative Testis

**Figure 6:**
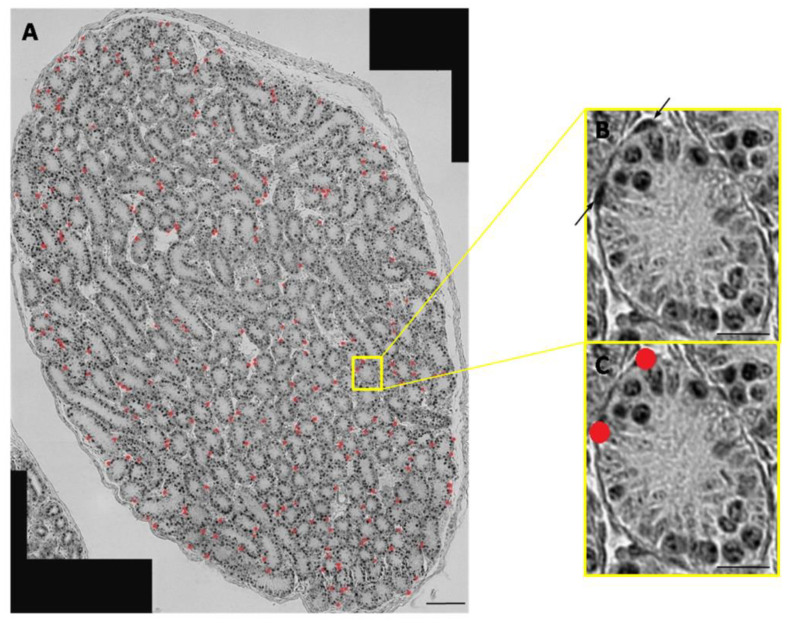
Visualization of proliferating peritubular cells in postnatal murine testis. Testicular tissue sections of wild-type mice were stained with a mAb against PCNA to determine the proliferating status of peritubular cells. (**A**) PCNA-positive peritubular cells are labeled with red dots in the cross section. Additionally, in an enlarged tubule, the same PCNA positive peritubular cells are labeled with (**B**) arrows or (**C**) red dots to illustrate the selection of positive cells. The PCNA-positive cells within the tubules were not considered in the study. Scale bars: (**A**) 100 µm; (**B**,**C**) 20 μm. Negative controls are provided in Supplemental Figure S1.

The absence of PKG1 and the decreased amount of calponin suggest that the differentiation of peritubular cells is negatively influenced in polySia knockout mice. Next, it was investigated, whether or not the postulated structural and functional deficits of peritubular cells coincide with higher cell proliferation, i.e., that a synthetic phenotype of SMCs predominates. Using the proliferation marker PCNA, peritubular cells of all tubules of a cross section were analyzed (Figure 6A). In addition to proliferating germ cells, PCNA-positive peritubular cells are clearly visible (arrows in Figure 6B). Afterwards, all positive peritubular cells were marked by red dots prior to the counting process (Figure 6C).

**Figure 7:**
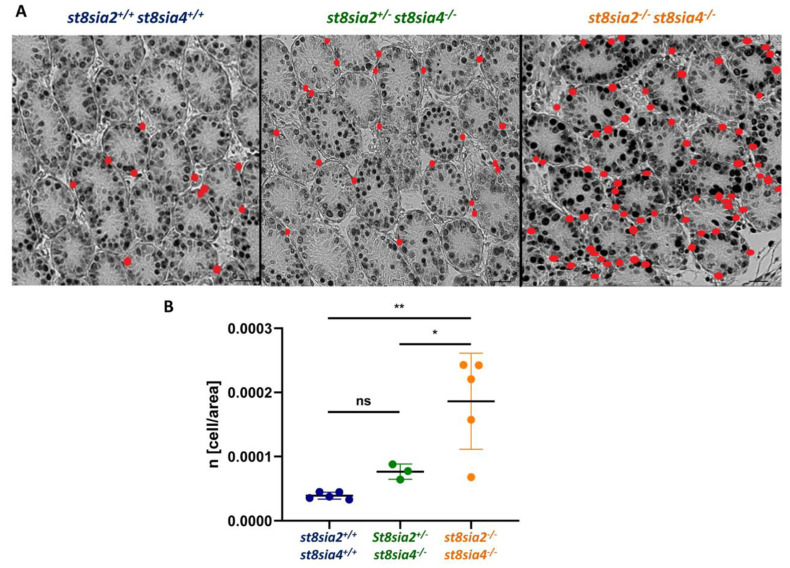
Investigation of proliferating peritubular cells in wild-type, *st8sia2^+/−^; st8sia4^−/−^* mice, and polySia-negative mice. (**A**) For the immunohistochemical visualization of proliferating peritubular cells, testicular sections of postnatal wild-type (*st8sia2^+/+^*; *st8sia4^+/+^*), *st8sia2^+/−^*; *st8sia4^−/−^*, and polysialyltransferase-deficient (*st8sia2^−/−^*; *st8sia4^−/−^*) mice were treated with a mAb against PCNA. The positive cells were labeled with red dots. Supplemental Figure 3 shows the same sections with smaller dots, which allow an additional evaluation of the peritubular cell-specific morphology. (**B**) Based on the immunohistochemical labeling of proliferating peritubular cells, the density of positive cells in the seminiferous tubules was calculated. The values (5 wild-type, 3 *st8sia2^+/−^; st8sia4^−/−^* and 5 double knockout animals; per animal 3 sections were analyzed) are summarized and standard deviation (SD) is displayed in addition to the means. Statistically significant differences are indicated: * *p* < 0.05; ** *p* < 0.01, ns: not significant. The PCNA-positive cells within the tubules were not considered in the study.

**Figure 8:**
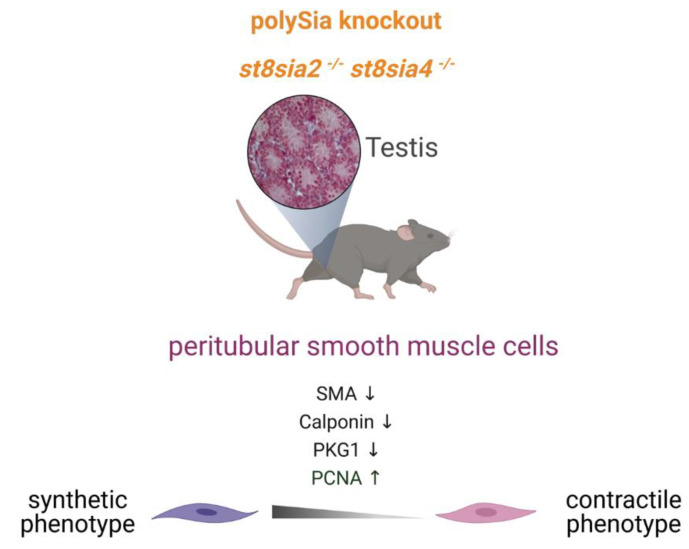
Summary of the observed phenotype. The loss of polySia leads to decreased protein levels of SMA, calponin and PKG1 in addition to increasing cell number of proliferating SMCs (PCNA positive) driving the differentiation of SMCs to a synthetic phenotype. Created with BioRender.com.

Based on this, the number of PCNA-positive peritubular cells per tubule area was determined in all three genotypes (Figure 7A and Supplemental Figure S3). The visual effect shown in Figure 7A is corroborated by statistical analysis, in which all groups are significantly different in the ratio of PCNA-positive peritubular cells to tubular area (Figure 7B). In *st8sia2^+/−^; st8sia4^−/−^* mice, a slight, but statistically not significant, increase of proliferating peritubular cells was visible. In polySia double knockout mice, however, there was a strong increase of proliferating peritubular cells compared to the wild-type. As suggested above for the observed loss of the contractile phenotype, the increased number of proliferating cells also implies a possible role of polySia during growth factor dependent processes. Interestingly, polySia-binding not only modulates the enrichment and presentation of growth factors to their respective receptors, but also shows protective effects against their proteolytic cleavage [34]. Thus, the half-life and binding efficiency of growth factors, such as bFGF, might be impaired. In sum, these results suggest a dedifferentiation in the absence of polySia, since also an increasing number of proliferating cells is an important characteristic of the synthetic phenotype of SMCs (Figure 8).

## 4. Conclusions

In sum, the outlined experiments demonstrate that in postnatal murine testes,

- polySia is detectable around seminiferous tubules.
- the strongest net-like intertubular polySia staining is detectable during the first week of development.
- the loss of polySia leads to a development of peritubular SMCs towards a synthetic phenotype (Figure 8).

Thus, polySia might influence the maintenance of a differentiated phenotype of non-vascular SMCs.

## Supplementary Materials

The following are available online at https://www.mdpi.com/article/10.3390/cells10061347/s1, Figure S1: Negative controls for immunohistochemistry. Figure S2: Immunohistochemical localization of SMA and CD31 in testicular tissue of postnatal mice. Figure S3: Labeling proliferating peritubular cells in wild-type, *st8sia2^+/−^; st8sia4^−/−^*, and polySia-negative mice.
