# Supplementary material for: The Loss of Polysialic Acid Impairs the Contractile Phenotype of Peritubular Smooth Muscle Cells in the Postnatal Testis
Source: Cells. 2021 May 29;10(6):1347. doi: 10.3390/cells10061347 (PMC8230264; doi:10.3390/cells10061347)
Supplement: Supplementary file 1 [file cells-10-01347-s001.zip › cells-1026103-supplementary.pdf]

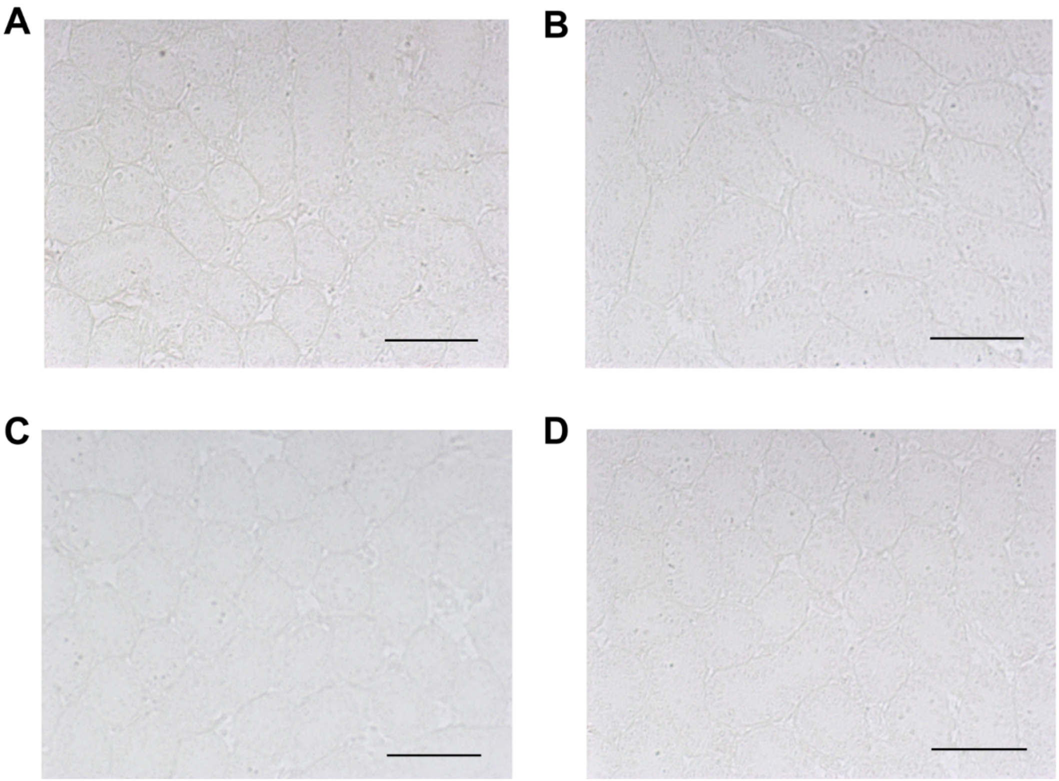

Supplemental Figure 1. Negative controls for immunohistochemistry. For all immunohistochemistry stainings ─ (a) SMA, (b) calponin, (c) PKG1, (d) PCNA ─ negative controls without the first antibodies were performed. In each case, no staining was visible. Scale bars: 100 μm.

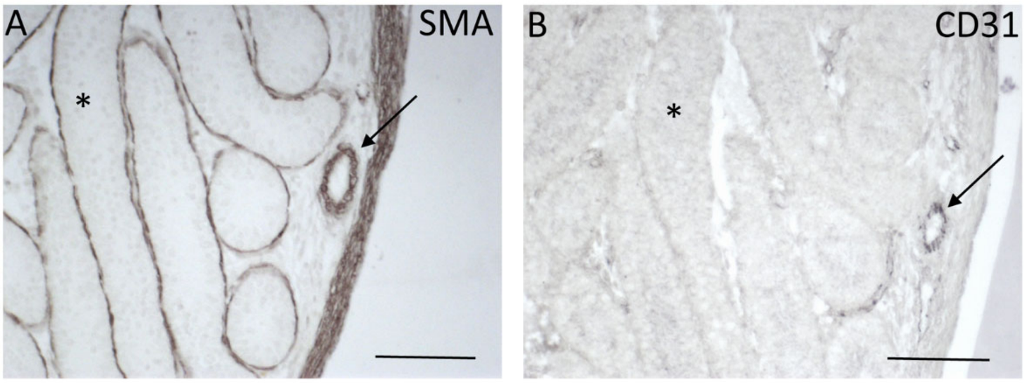

Supplemental Figure 2. Immunohistochemical localization of SMA and CD31 in testicular tissue of postnatal mice. Serial testicular tissue sections of postnatal wild-type mice were stained with mAbs against (a) SMA and (b) CD31. Arrows (blood vessels) and asterisks (tubules) indicate that the same structures are visible in the displayed serial sections. SMA immunostaining marks all contractile elements of the section (tunica albuginea, seminiferous tubules, blood vessels), whereas the endothelial marker CD31 visualized only blood vessels. Scale bars: 100 μm.

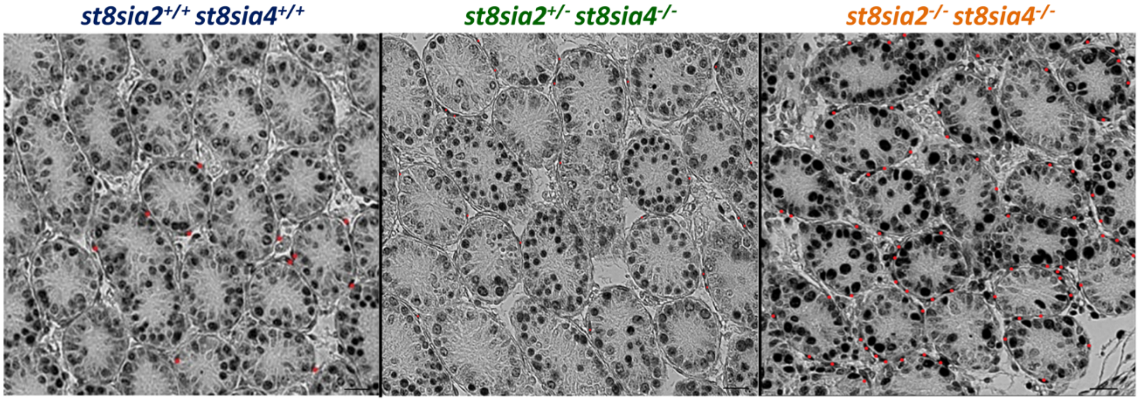

Supplemental Figure 3. Labeling proliferating peritubular cells in wild-type, *st8sia2*$^{+/-}$; *st8sia4*$^{-/-}$, and polySia-negative mice. Proliferating peritubular cells in testicular sections of postnatal wild-type (*st8sia2*$^{+/+}$; *st8sia4*$^{+/+}$), *st8sia2*$^{+/-}$; *st8sia4*$^{-/-}$, and polysialyltransferase-deficient (*st8sia2*$^{-/-}$; *st8sia4*$^{-/-}$) mice were visualized with a mAb against PCNA. The same tissue areas are also shown in Figure 6. However, smaller red dots are used to allow an evaluation of the PCNA staining in single peritubular cells. Scale bars: 20 µm. The PCNA-positive cells within the tubules were not considered in the study.
